# epiCOLOC: Integrating Large-Scale and Context-Dependent Epigenomics Features for Comprehensive Colocalization Analysis

**DOI:** 10.3389/fgene.2020.00053

**Published:** 2020-02-12

**Authors:** Yao Zhou, Yongzheng Sun, Dandan Huang, Mulin Jun Li

**Affiliations:** ^1^ Department of Pharmacology, Tianjin Key Laboratory of Inflammation Biology, School of Basic Medical Sciences, National Clinical Research Center for Cancer, Tianjin Medical University Cancer Institute and Hospital, Tianjin Medical University, Tianjin, China; ^2^ Collaborative Innovation Center of Tianjin for Medical Epigenetics, Tianjin Key Laboratory of Medical Epigenetics, Tianjin Medical University, Tianjin, China

**Keywords:** colocalization, epigenomics and epigenetics, functional annotation analysis, genetic variants, cell type specific, web server

## Abstract

High-throughput genome-wide epigenomic assays, such as ChIP-seq, DNase-seq and ATAC-seq, have profiled a huge number of functional elements across numerous human tissues/cell types, which provide an unprecedented opportunity to interpret human genome and disease in context-dependent manner. Colocalization analysis determines whether genomic features are functionally related to a given search and will facilitate identifying the underlying biological functions characterizing intricate relationships with queries for genomic regions. Existing colocalization methods leveraged diverse assumptions and background models to assess the significance of enrichment, however, they only provided limited and predefined sets of epigenomic features. Here, we comprehensively collected and integrated over 44,385 bulk or single-cell epigenomic assays across 53 human tissues/cell types, such as transcription factor binding, histone modification, open chromatin and transcriptional event. By classifying these profiles into hierarchy of tissue/cell type, we developed a web portal, epiCOLOC (http://mulinlab.org/epicoloc or http://mulinlab.tmu.edu.cn/epicoloc), for users to perform context-dependent colocalization analysis in a convenient way.

## Introduction

The epigenome, beyond genome sequence, has been increasingly recognized as key component in the gene regulation to drive certain biological processes and associate with many human diseases (Lawrence et al., 2016; Dor and Cedar, 2018; Feinberg, 2018). In the past decades, high-throughput epigenomic sequencing assays have profiled large numbers of functional elements across numerous human tissues/cell types, such as histone modification, DNA methylation, open chromatin, transcription factor binding site (TFBS), etc. The International Human Epigenome Consortium (IHEC) project (Bujold et al., 2016) have been initialized, across different countries and consortiums, to coordinate the production of reference maps of human epigenomes for key cellular states relevant to health and diseases. These unprecedented growths of epigenetic profiles and following comprehensive analysis of tissue/cell type-specific epigenomes will ultimately lead to a better understanding of how human population and genome function are shaped in response to the environment (Egtex, 2017).

To facilitate convenient and accurate utilization of increasing volume of epigenomic data, several commonly-used resources have uniformly processed raw profiles and made them easily accessible, including ENCODE (Consortium, 2012), Roadmap Epigenomics (Roadmap Epigenomics et al., 2015), Blueprint Epigenome (Stunnenberg et al., 2016) and CistromeDB (Mei et al., 2017; Zheng et al., 2019). Furthermore, comprehensive epigenomics accumulation has motivated novel computational methods of modelling functional elements across many tissues/cell types, such as ChromHMM (Roadmap Epigenomics et al., 2015) and Segway (Libbrecht et al., 2019). Therefore, integrating such large-scale and context-dependent epigenomics features for novel biological findings is in urgent demand (Dozmorov, 2017; Cazaly et al., 2019). To this end, colocalization analysis was frequently used to study the interplay of various functional elements in different biological processes and conditions, where potential enrichment of a given genomic/epigenomic profile in pre-defined dataset could be drawn from the global perspective (Kanduri et al., 2019). Integrated with large-scale tissue/cell type-specific epigenomics data, colocalization analysis provides a powerful avenue to investigate biological relations and cell type specificities, such as identifying co-occurrence of transcription regulators (Yan et al., 2013) and inferring causal tissues/cell types from disease-associated variants identified by genome-wide association study (GWAS) (Farh et al., 2015).

Many colocalization tools have been developed by holding diverse assumptions and background models to assess the significance of enrichment. For instances, GSuite HyperBrowser is a web-based tool that performs colocalization analysis using either analytical approaches or Monte Carlo simulations (Simovski et al., 2017). LOLA utilizes Fisher's exact test based on universe regions to inspect enrichment and provides a web-based portal LOLAweb (Sheffield and Bock, 2016; Nagraj et al., 2018). GoShifter (Trynka et al., 2015) and GARFIELD (Iotchkova et al., 2019), which were implemented into standalone tools, specifically quantify enrichment of overlaps between GWAS variants and genomic annotations by considering linkage disequilibrium (LD). To overcome the discordant enrichment among exiting methods, Coloc-stats integrates multiple colocalization analysis tools in a single web interface (Simovski et al., 2018). This integrated system serves as a one-stop shop for performing comprehensive colocalization analysis and asseses the consistency of the conclusions across seven different methods. However, some critical issues remain unaddressed. First, existing tools only provide limited pre-defined sets for genomic features in different biological domains. Current web-based tools, such as GSuite HyperBrowser, GenomeRunner (Dozmorov et al., 2016) and LOLAweb, only incorporate a small number of epigenomic profiles from ENCODE, Cistrome and other specific annotation datasets, which restrict the broader applications of online colocalization analysis. Second, the descriptions of tissue and cell type information are disordered and only based on free text, making current tools unable to properly classify or group tissues/cell types to inspect the specificity of enrichment. Therefore, a uniform human tissue/cell-type definition is needed. Furthermore, the growing volume of epigenomic profiles on extensive tissues/cell types, collection and integration of these genomic features require a great effort to download. Most colocalization web tools are time-consuming for features intersection and background generation when dealing with such accumulating data scale. To ease the comprehensive colocalization analysis for biologists and geneticists, a faster and versatile online platform would be welcome.

For this study we comprehensively collected and integrated over 44,385 bulk or single cell epigenomic profiles across 53 human tissues/cell types. By classifying and mapping these profiles into hierarchy of tissue/cell type, we developed a web portal, epiCOLOC, for users to perform context-dependent colocalization analysis in a convenient way. We leveraged a recent ultrafast genomics search engine, GIGGLE, to identify and prioritize the enrichment of genomic loci shared between query features and our pre-defined epigenomic interval files (Layer et al., 2018). epiCOLOC equips many visualization functions and is freely available at http://mulinlab.org/epicoloc or http://mulinlab.tmu.edu.cn/epicoloc.

## Epigenomic Profiles Integration and Processing

### Data Collection

**Figure 1:**
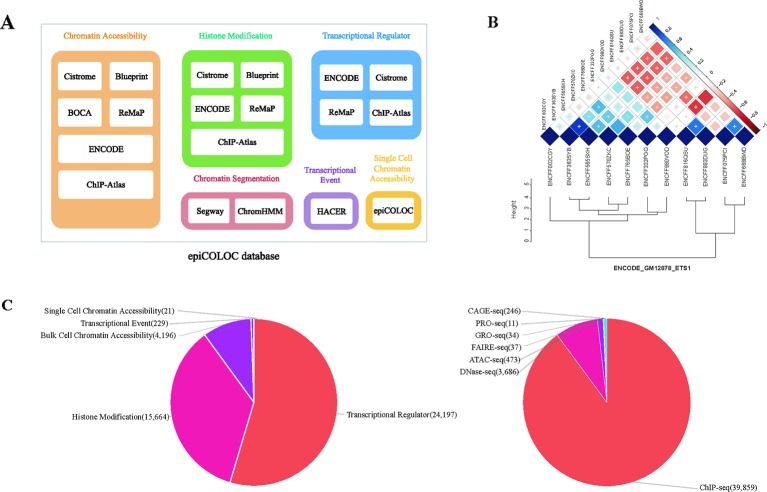
The overview of epiCOLOC design and datasets. **(A)** The source schema of epiCOLOC data collection. **(B)** An example to illustrate outlier profiles removing. **(C)** The summary of data types in the current version of epiCOLOC.

We collected human genomic and epigenomic data from various public resources including ENCODE (Consortium, 2012), Roadmap Epigenomics (Roadmap Epigenomics et al., 2015), Cistrome (Mei et al., 2017), ReMap (Cheneby et al., 2018), ChIP-Atlas (Oki et al., 2018), DeepBlue (Albrecht et al., 2017), BOCA (Fullard et al., 2018), TCGA (Corces et al., 2018) and HACER (Wang et al., 2019) (**Supplementary Table 3**). According to data sources and corresponding attributes, we classified collected features into following categories: 1) Transcriptional regulator, which incorporates ChIP-seq profiles of large number of transcriptional factors and chromatin remodelers; 2) Histone modification, which incorporates ChIP-seq profiles of different histone modifications; 3) Chromatin accessibility, which contains DNase-seq, ATAC-seq and FAIRE-seq profiles of open chromatin; We also curated several single cell ATAC-seq assays in this category; 4) Transcriptional event, which contains CAGE-seq, GRO-seq and PRO-seq profiles of nascent transcription signals; 5) Chromatin segmentation, which introduces tissue/cell type-specific chromatin states predicted by ChromHMM and Segway (**Figure 1A** and **Supplementary Table 1**). In order to improve accuracy and robustness of epiCOLOC backend database, we removed low-quality profiles according to the quality control scheme provided in the original resource. For example, we removed ChIP-seq data not passing two Cistrome quality metrics, including fraction of reads in peaks, and sufficient number of peaks with good enrichment. We also excluded ENCODE profiles with error audit flags, such as extremely low read length, not tagged antibody, etc. Current epiCOLOC database covers 1,631 chromatin markers, which comprises 88 histone modifications, 1,538 transcriptional regulators, open chromatin and transcriptional event.

### Data Processing

#### Tissue Organization and Mapping

We mapped cell lines to tissues by accounting for some auxiliary information from original epigenomic studies and several standards from GTEx (Consortium et al., 2017), Expression Atlas (Papatheodorou et al., 2018), Cellosaurus (Bairoch, 2018), ATCC (www.atcc.org), and BRENDA Tissue Ontologies (www.ebi.ac.uk/ols/ontologies/bto), yielding 53 main human tissues in total. For some main tissues that contain multiple well characterized components or some cell lines that cannot simply map to specific main tissues, we set independent terms in tissue set and finally generated 137 sub-tissues (**Supplementary Table 2**). We then manually mapped tissue/cell type name of each profile to our uniformly defined tissue set.

#### Cell Type Mapping

To reduce the complexity of cell type description in our collected epigenomic profiles, we performed cell type mapping using Cellosaurus that collected almost all cell line synonyms in a reference database (Bairoch, 2018). We acquired the Cellosaurus accession numbers and corresponding synonyms for all recorded cell lines, and assigned uniform synonyms identifiers to epigenomic profiles, which greatly reduces the heterogeneity of cell type descriptions. For cancer cell types mapping, we borrowed DepMap which provides standard terms for over thousands of cancer cell lines and organoid models (Van Der Meer et al., 2019). Since DepMap provides Cellosaurus accession numbers, we were able to easily map cancer cell lines to consistent reference.

#### Profile Grouping

Since the epigenomic data were generated by different laboratories or produced using different protocols, replicates and analysis methods among collected sources, we sought to identify profiles describing similar biological processes in each source. We grouped all collected profiles according to source + assay type + tissue/cell type + biological target, and assigned unique group identifiers to them.

#### Outlier Profiles Removal

To further ensure informative profiles in each group, we designed a strategy to eliminate potential outlier profiles that may deviate from underlying biological process of the group (**Supplementary Methods**). For each group with at least three profiles, we first constructed a pair-wise similarity matrix for all profiles based on GIGGLE combo score (Layer et al., 2018). Then, hierarchical clustering was used to cluster these profiles based on Euclidean distance and the optimal number of clusters was automatically determined by inconsistency coefficient method (Zahn, 1971). Furthermore, we only retained profiles within the largest cluster as representatives in this group. For example, we identified that four outlier profiles among 11 ETS1 ChIP-seq peak profiles in GM12878 cell line, and excluded them in the colocalization analysis (**Figure 1B**).

### epiCOLOC Web Tool Implementation

The current version of epiCOLOC incorporates 44,385 tissue/cell type-specific functional profiles from 44,364 bulk-cell studies and 21 single-cell studies after quality control (**Supplementary Table 4**). Most of these profiles (89.8%) are derived from ChIP-seq for transcription regulators and histone modifications, while, 9.5% profiles came from DNase-seq and ATAC-seq for chromatin accessibility (**Figure 1C**).

### Colocalization Method

To achieve a fast and efficient colocalization based on high volume epigenomic features, we embedded a genomic feature search engine, GIGGLE, into epiCOLOC web server (Layer et al., 2018). GIGGLE uses Fisher's exact test and odds ratio of “observed” versus “expected” to measure enrichment between query features and pre-indexed genomic intervals. It also creates a combination score called GIGGLE combo score, which is the product of -log10(Fisher's exact test *P*-value) and log2(odds ratio). Given thousands of epigenomic profiles in epiCOLOC database, GIGGLE can significantly reduce the running time from hours to minutes. For example, epiCOLOC takes about 6 minutes to finish colocalization analysis on transcriptional regulator profiles of all blood cells for a set of 10k intervals (randomly generated genomic intervals with varying length). For each profile group, we calculated median score to represent group-level enrichment. With the aid of efficient colocalization strategy, epiCOLOC tries to provide powerful context-specific epigenomic evidences, leading to novel biological problems identification, such as “Are two transcription factors (TFs) colocalized and forming cooperation” or “Are the query variants/intervals enriched in chromatin open regions of specific tissues?” or “Are the query variants/intervals overlap with transcribed enhancers regions more than would be expected by chance?” More biological examples can be found in our website http://mulinlab.org/epicoloc/Introduction/#Biological-examples.

### Web Interface and Usage

epiCOLOC was implemented in a web-based tool with built-in large-scale and context-dependent epigenomic annotations. The epigenomic profiles were indexed using GIGGLE. The web server was developed by Python, jQuery, igv.js, amcharts.js and related JavaScript modules.

#### Querys

epiCOLOC accepts two types of genomic format: BED-like format and VCF-like format. Both plain text and uploaded file of regions of interest (ROIs) or variant positions are well supported. Uploaded file can be BED or VCF text file or compressed gzip file (<20Mb).

#### Options

epiCOLOC provides several options for users to customize colocalization analysis, including 1) select tissues (53 tissues/137 sub-tissues); 2) select profile categories (Transcriptional regulator, Histone modification, Chromatin accessibility, Transcriptional event, Chromatin segmentation); 3) change human genome assembly (GRCh37 and GRCh38); 4) define background genome size (3,095,677,412 for GRCh37 and 3,088,269,832 for GRCh38 in default); 5) set maximal interval length (500bp in default, and ROIs which exceed maximum length will be removed); 6) set extended length on both sides (no extension by default); 7) set central window size (cut the central area of genomic intervals, no central window by default).

#### Job Submission

Once submitted, the job will be sent to the backend of the web server for colocalization analysis. epiCOLOC displays a progress bar to track the execution status. It allows job retrieval by searching for the job ID in the home page, or by using a fixed URL (http://mulinlab.org/epicoloc/<jobid>) to check results directly, or through email notification.

#### Results Visualization

**Figure 2:**
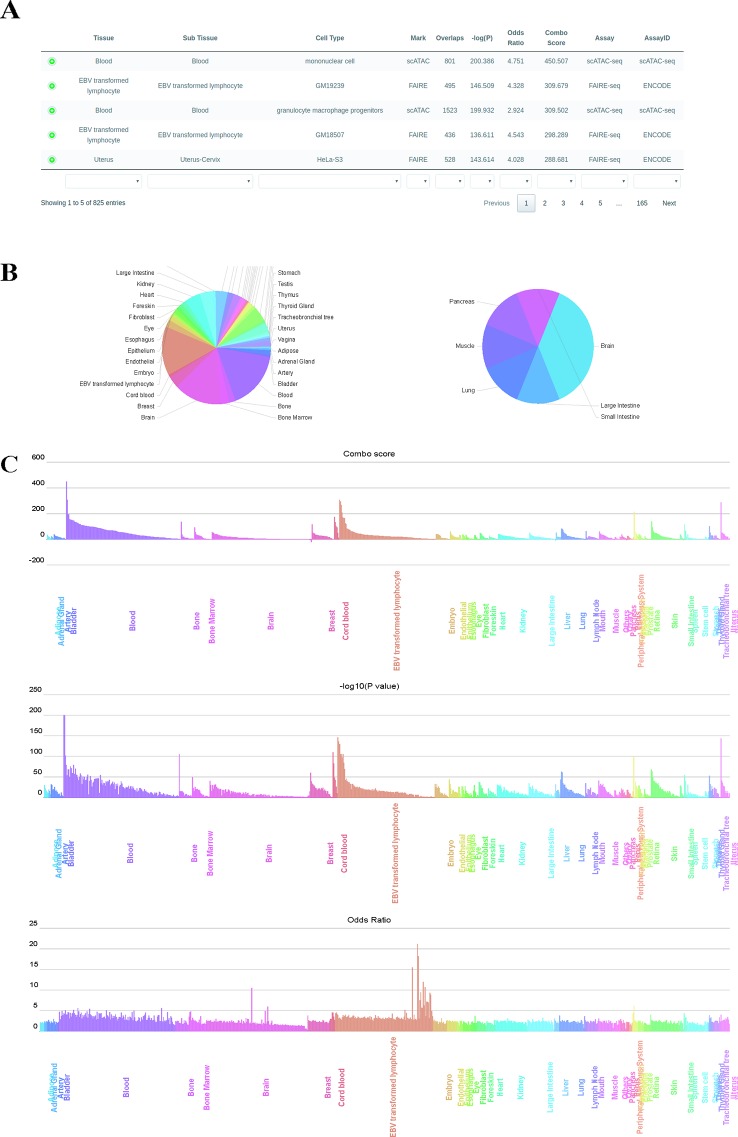
Results page of epiCOLOC. Colocalization result for IBD GWAS variants in open chromatin regions, **(A)** Prioritization table. **(B)** Pie chart that depicts the number of significant enriched or depleted profiles in each tissue. **(C)** Bar plots that display ordered combo score, *P*-value, odds ratio in tissue-wise manner.

We used GIGGLE combo scores to prioritize colocalization results. Higher combo score indicates better enrichment on a specific profile, while negative combo scores suggest depleted enrichment (**Supplementary Figure 1**). Users can inspect and visualize the results in four different manners: 1) Prioritization table, which shows statistics metrics of colocalization including combo score, Fisher's exact *P-*value, odds ratio, the number of overlaps and extra information of enriched profiles (**Figure 2A**); 2) Tissue-wise pie charts for enrichment and depletion, which depict the per tissue proportion in all enriched (positive combo score) or depleted (negative combo score) profiles (**Figure 2B**). Users can click the slice of each tissue in the pie chart to see detailed sub-tissue results; 3) Tissue-wise bar plots, which display the representative enriched or depleted profiles in each tissue (**Figure 2C**). The user can search, scroll, zoom and hover over the bar plot to get detailed information of enrichment (only assay IDs for the best profiles in each group are displayed in hover tooltip). Once the label under the tissue-wise bar plotsis clicked, cell type-wise bars which depict enrichment patterns for the top 20 enriched cell types appear in a pop-up window. 4) The IGV dashboard displays relative genomic location for queries genomic intervals and top five enriched profiles in colocalization analysis.

#### Download

epiCOLOC allows users to download colocalization results in csv format and result figures in png, jpg or pdf formats.

## Case Studies and Evaluations

By integrating large-scale tissue/cell type-specific epigenomic profiles, epiCOLOC could be used to investigate many biological questions. Here, we used several examples to demonstrate the performances and potential usages of epiCOLOC.

To identify potential disease-relevant genomic features and tissues using GWAS variants, we first performed colocalization analysis on disease-associated variants for inflammatory bowel disease (IBD) (Liu et al., 2015) to test the tissue-specific enrichment. Using chromatin accessibility features, we found that IBD GWAS variants (*P*-value < 5E-8) were significantly enriched in blood tissue, where open chromatin profiles on monocyte, lymphocyte and granulocyte macrophage progenitor received highest enrichment scores. (**Figure 2**, and also see colocalization result from: http://mulinlab.org/epicoloc/results/bc2fa49a-6dfa-40f1-bb61-1349c9118168). This result was consistent with GARFIELD results using functional annotations from ENCODE and Roadmap Epigenomics (Iotchkova et al., 2019). We then used coronary artery disease (CAD) GWAS variants (*P*-value < 5E-8) to perform colocalization in open chromatin regions (Van Der Harst and Verweij, 2018). Consistent with GARFIELD reports, we observed that most of tissues showed similar enrichment patterns, without distinct tissue specificity at open chromatin (http://mulinlab.org/epicoloc/results/63b0cd1b-f22f-43dd-9452-fdea114f6c3d). However, when using fine-mapped CAD variants, we observed several highly enriched signals in tissues like liver and artery blood vessel (http://mulinlab.org/epicoloc/results/04bf79a8-f7cd-4960-913e-5c5c84c05753), implying that the importance of selecting informative ROIs before colocalization analysis.

Next we sought to demonstrate that whether epiCOLOC could be used to identify potential cooperative factors for given TF. Transcription factor 7-like 2 (TCF7L2), a TF in the Wnt-signaling pathway, has been proven to play a central role in coordinating the expression of proinsulin and forming mature insulin (Zhou et al., 2014). TCF7L2 binding sites had been reported to colocalize with HNF4alpha and FOXA2 in HepG2 cell (Frietze et al., 2012). We hence used TCF7L2 ChIP-seq in HepG2 to perform colocalization analysis using epiCOLOC. In our colocalization results, TCF7L2 ChIP-seq peaks were significantly enriched in EP300, CREM, SP1, FOXA2 and HNF4alpha ChIP-seq profiles in various tissues/cell types (http://mulinlab.org/epicoloc/results/d736578a-59a4-4160-a6fe-1a9c420c4adf). Furthermore, we used two motif finding tools, PscanChIP (Zambelli et al., 2013) and HOMER (Heinz et al., 2010), with the same query input to investigated enriched TF motifs. We found that TF motifs including HNF4alpha, FOXA2, TCF7, GATA4, FOXP1, FOXA1, FOXK2 and FOXO3 can be simultaneously identified among two motif finding tools and our epiCOLOC, which also validates the efficacy of our tool.

## Discussion

In this study, we have integrated a comprehensive and tissue/cell type-specific epigenomics profiles database. With strict pre-processing, quality control and tissue mapping, we established a user-friendly web portal, epiCOLOC, which to perform fast and context-dependent colocalization analysis; and provide a series of visualization functions to interpret results; and significantly distinguish between existing web-based tools (**Supplementary Table 5**). In the applied examples, we demonstrated the accuracy and practicality of epiCOLOC in identifying causal tissues/cell types from GWAS disease-associated variants and inferring co-occurrence of transcription regulators.

There are some limitations in this work which deserve optimization in our future works. First, the statistical assumption of GIGGLE is simple and could be sub-optimal in several cases. We strongly recommend users to prioritize results by combo score and set stringent thresholds. As observed from the combo scores distribution when *P*< = 0.05 using query intervals that randomly generated in genome (**Supplementary Figure 2**), we propose to use an empirical combo score cutoff, 5 for enrichment and -2 for depletion, as advisable criteria to further filter enrichment or depletion results. Although GIGGLE can greatly speed up colocalization analysis, as compared with GenomeRunner (Dozmorov et al., 2016) and LOLAweb (Nagraj et al., 2018), it limits the usage of user-specific background of genomic regions and the analysis of multiple genomic intervals. Second, although epiCOLOC is applicable to perform colocalization analysis using genetic variants, but it cannot account for LD and allele frequency. Third, there are uneven epigenomic profiles for different tissues/cell types. It may potentially affect the robustness of colocalization when applying epiCOLOC to the tissues/cell types having fewer data available, and it also cannot determine the missing enrichment for tissues/cell types lacking sufficient data. In addition, single-cell technologies, such as single-cell ATAC-seq and single-cell ChIP-seq (Grosselin et al., 2019), have been developed to analyze genome-wide epigenomic features. Such approaches pave the way to study the role of epigenetic heterogeneity in many biological conditions and will be largely incorporated into epiCOLOC in the next stage. Recently, a novel algorithm named Augmented Interval List (AIList) (Feng et al., 2019), which introduces a new data structure and provides a significantly improved fundamental operation for highly scalable genomic data analysis. This method together with upcoming large-scale genomic features will be added in the epiCOLOC future updates.

## Data Availability Statement

Publicly available datasets were analyzed in this study. This data can be found from ENCODE, Roadmap Epigenomics, etc and also related sources has been listed here: http://mulinlab.org/epicoloc/Introduction/.

## Author Contributions

ML designed and guided the study, YZ, YS and DH developed the tool, YZ and ML wrote the manuscript.

## Funding

This work was supported by grants from the National Natural Science Foundation of China 31871327, 31701143 (ML), Natural Science Foundation of Tianjin 18JCZDJC34700, 19JCJQJC63600 (ML). We also appreciate all tool and resource providers.

## Conflict of Interest

The authors declare that the research was conducted in the absence of any commercial or financial relationships that could be construed as a potential conflict of interest.
