# Supplementary material for: epiCOLOC: Integrating Large-Scale and Context-Dependent Epigenomics Features for Comprehensive Colocalization Analysis
Source: Front Genet. 2020 Feb 12;11:53. doi: 10.3389/fgene.2020.00053 (PMC7029718; doi:10.3389/fgene.2020.00053)
Supplement: Supplementary file 1 [file DataSheet_1.docx]

**Supplementary Figures**


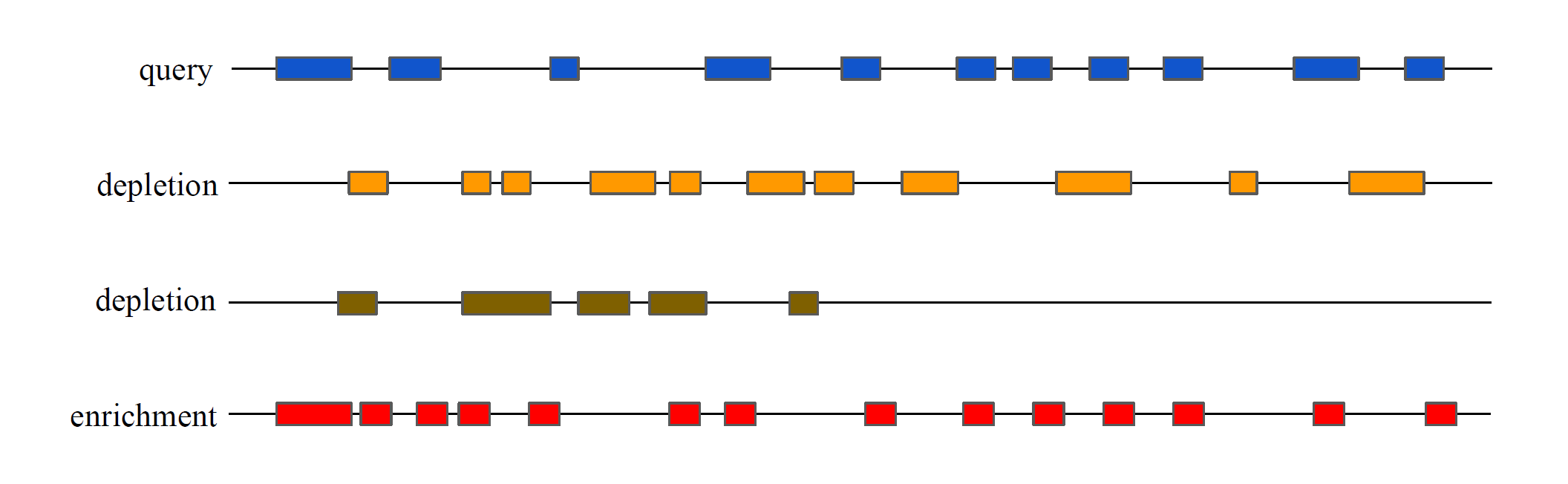


Figure S1: illustrative diagram of depletion and enrichment scenarios


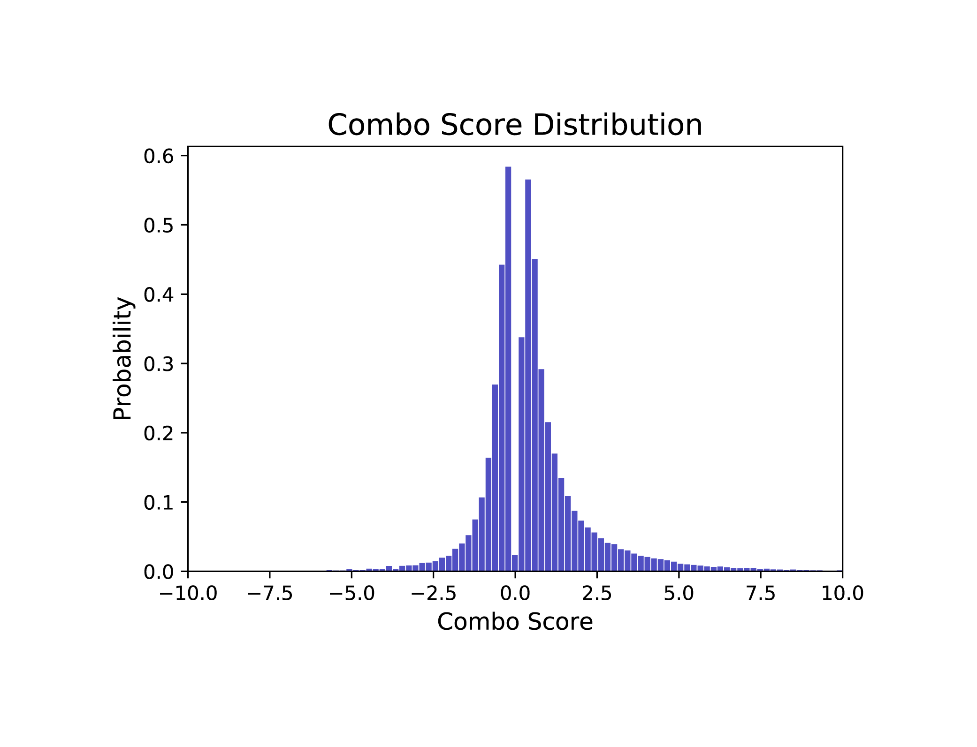


Figure S2: distribution of GIGGLE combo score using query intervals that were randomly generated in the human genome

**Supplementary Methods**

For each group with at least three profiles, we first constructed a pair-wise similarity matrix for all profiles based on GIGGLE combo score (Layer et al., 2018).

We calculated the Euclidean distance between every pair of objects in the similarity matrix to describe their dissimilarities, and then group the objects into a binary hierarchical cluster tree based on Ward Linkage algorithm. Inconsistency method computes inconsistency coefficient by comparing each cluster merge’s height *h* to the average height *avg* and normalizing it by the standard deviation *std* formed over the depth *d* previous levels.

$$inconsistency coefficient=(h-avg)/std$$

The larger the coefficient, the greater the difference between the objects connected by the link.

In the practical processes, we use 5 as depth *d*, and formed flat clusters from the hierarchical clustering defined by the linkage matrix under the criterion that if a cluster node and all its descendants have an inconsistent value less than or equal to 2 then all its leaf descendants belong to the same flat cluster. Finally, we assign the flat clusters to profiles and retained profiles within the largest cluster as representatives to this group.

Layer, R.M., Pedersen, B.S., Disera, T., Marth, G.T., Gertz, J., and Quinlan, A.R. (2018). GIGGLE: a search engine for large-scale integrated genome analysis. *Nat Methods* 15**,** 123-126.
